# COVID-19 and psychosis, depression, obsession and quality of life in Lebanese patients with schizophrenia: Any changes after 5 months of quarantine?

**DOI:** 10.1186/s40359-022-00750-7

**Published:** 2022-02-19

**Authors:** Chadia Haddad, Joseph E. Dib, Nadine Akl, Souheil Hallit, Sahar Obeid

**Affiliations:** 1 Research Department, Psychiatric Hospital of the Cross, Jal Eddib, Lebanon; 2 INSPECT-LB (Institut National de Santé Publique, d’Épidémiologie Clinique et de Toxicologie-Liban), Beirut, Lebanon; 3 Division of Psychiatry and Clinical Psychology, School of Medicine, University of Nottingham, Nottingham, UK; 4 School of Medicine and Medical Sciences, Holy Spirit University of Kaslik, P.O. Box 446, Jounieh, Lebanon; 5 Social and Education Sciences Department, School of Arts and Sciences, Lebanese American University, Jbeil, Lebanon

**Keywords:** Covid-19, Quarantine, Depression, Quality of life, Obsession, Schizophrenia

## Abstract

**Background:**

Previous research revealed an absence of any previous studies reporting the impact that pandemics may have on psychotic symptomology, nor on the physical health of people with psychosis in response to the epidemics of the COVID-19. The direction of the impact of the COVID-19 on schizophrenia is unknown, as the risk of infection could vary from patients to patients according to clinical comorbidities, cognitive impairment, acute symptoms, and family support. To the best of our knowledge, no study has provided details on the variation of symptoms in patients with schizophrenia during the quarantine of COVID-19 outbreak. Therefore, the primary objective of the study is to investigate the variation of psychotic symptoms, depression, obsession and quality of life in patients with schizophrenia before and after 5 months of quarantine and evaluate factors associated with these variations during the quarantine period.

**Methods:**

A cross-sectional study was performed on a sample of 190 chronic patients institutionalized for schizophrenia for more than 1 year at the Psychiatric Hospital of the Cross. The baseline assessment was done in December 2019; the second assessment was done in August 2020 (5 months after the lockdown).

**Results:**

Getting updates about the coronavirus minimally, some and most of the times were significantly associated with a decrease in positive psychotic and psychopathologic symptoms 5 months after quarantine compared to before it. Practicing religiosity some and all the time versus not was significantly associated with a decrease in negative, psychopathology symptoms and total PANSS score after 5 months of quarantine compared to before it. Finally, female gender (B = 1.77) was significantly associated with an increase in the WHO Domain 3 score (better social relations) after 5 months of quarantine compared to before it.

**Conclusion:**

Patients with schizophrenia fare better symptomatically after 5 months of quarantine if they receive constant updates about COVID-19 and if they tended to practice religiosity.

## Background

The Coronavirus disease 2019 (COVID-19) outbreak is a global pandemic that originated in China in December 2019, and had spread all over the world by March 2020 [1, 2]. Presently, COVID-19 has infected 279,114,972 individuals worldwide and has led to 5,397,580 deaths as reported by the World Health Organization (WHO) as of December 23, 2021 [3]. Although the production of a vaccine is ongoing, public health strategies such as quarantine and social distancing have been implemented in almost every country to prevent the spread of the disease. Social distancing can be the most effective way to prevent the virus from spreading. However, this measure is associated with a variety of psychological effects such as fear and anxiety [4, 5]. Such impacts can vary across populations particularly in patients with severe mental illness such as schizophrenia.

Psychiatric patients are among the most vulnerable populations affected by the COVID-19 pandemic [6]. Globally, individuals with severe mental illnesses live depending on which region of the world they reside in and the guidelines, healthcare and other facilities available [7]. In a recent study done in China among seventy-six psychiatric patients and 109 healthy control subjects have found a negative psychological impact on psychiatric patients during the COVID-19 epidemic with strict lockdown measures [8]. Also, psychiatric patients were significantly more likely to report worries about their physical health, anger, impulsivity, and suicidal ideation [8]. Most individuals with severe mental illnesses in Lebanon live in institutional environments such as psychiatric hospitals and nursing homes [9, 10]. These population are at increased risk of infectious disease outbreaks due to shared spaces and an overcrowding environment [11, 12]. In addition, the risk of infection with COVID-19 is higher among this high risk population as it has been found that these individuals find it difficult to follow appropriate infection control measures due to the psychiatric and behavioral factors relating to cognitive impairment and poor insight [11, 13]. People with psychotic symptoms may be less motivated to adhere to infection control and physical distancing measures and appear to have deficits in hygiene practices [14, 15]. These behaviors could certainly increase vulnerability to respiratory diseases. In addition, the psychiatry units are not designed to contain dangerous highly infectious diseases, staff and patients do not usually wear protective equipment, and psychiatric patients may find it difficult to socially distance as they live in the same environment [16]. Moreover, the treatment of psychiatric illness requires social interaction as patients attend groups and occupational therapy sessions, and live in a community environment [17]. Consequently, poor self-care and impaired judgment which are two characteristics found in schizophrenia, may hinder compliance with health guidelines and place patients, their families and health professionals at risk [17].

Social isolation and physical distancing imposed by the quarantine measures may further increase stress and may worsen physical and mental health in people with schizophrenia affecting their overall quality of life [18]. In a study done by Wang et al. among 2266 respondents from two countries (1056 Poles and 1210 Chinese) have found that hygiene practice was associated with lower anxiety scores in Polish and Chinese respondents however, the use of face mask and satisfaction with health information were not associated with mental health parameters in Poles and Chinese [19]. A nationwide lockdown in Lebanon was imposed by the Lebanese government to face the COVID-19 pandemic that lasted from March until June 2020; it would help reduce the contagious spread as well as ease pressure off the medical services. The entire educational establishment was closed in addition to restaurants and touristic attractions, with social gatherings being banned.

The support obtained by their families and friends are reduced due to physical distancing therefore generating considerable distress, both for patients and caregivers hence why depression levels were closely monitored in this study [20, 21]. The negative psychological effects include confusion, anger and post-traumatic stress symptoms that consequently might increase the risk of psychiatric exacerbation, including acute agitation, psychosis, mania or severe depression [18]. Moreover, the risk of psychotic symptoms increasing is high due to a lack of access to routine psychosocial and medication interventions [17]. In addition, obsession symptoms might exist due to the increased paranoia of contracting the disease by being in close contact with other people [17]. In a rapid review, Brown et al. [16] found an absence of any previous studies reporting the impact that pandemics may have on psychotic symptomology, nor on the physical health of people with psychosis in response to the epidemics of the COVID-19. The direction of the impact of the COVID-19 on schizophrenia is unknown, as the risk of infection could vary from patients to patients according to clinical comorbidities, cognitive impairment, acute symptoms, and family support. To the best of our knowledge, no study has provided details on the variation of symptoms in patients with schizophrenia during the quarantine of COVID-19 outbreak. Therefore, the primary objective of the study is to investigate the variation of psychotic symptoms, depression, obsession and quality of life in patients with schizophrenia before and after 5 months of quarantine and evaluate factors associated with these variations during the quarantine period.

## Methods

### Study design and participants

In this cross-sectional study, patients were recruited from the inpatient services of the Psychiatric Hospital of the Cross (HPC), which is the largest psychiatric hospital in Lebanon and in the Middle East [22]. The HPC is located in Mount Lebanon region near Beirut the capital city of Lebanon with a capacity around 900 psychiatric beds. It has responded rapidly to the COVID-19 outbreak in the country having established enclosed measures, such as temporarily stopping inpatient admission and prohibiting visits. Furthermore, the conditions of confinement at the hospital included the absence of outing for home leave. Patients have been left in enclosed environment far from their parents and relatives, with a possible contact with them made available only via phone call. Within the hospital, patients are allowed to leave their rooms and socialize together. Protective facemasks were worn by the patients when leaving the ward to other sections of the hospital due to medical related reasons. In addition, the staff complied with safety measures by wearing a facemask during their shifts. It is important to note, however, that no personal full body protective equipment was worn by the staff due to having no COVID-19 cases being declared in the hospital at the time. The baseline assessment was done in December 2019; the second assessment was done in August 2020 (5 months after the lockdown), among patients with a diagnosis of schizophrenia (confirmed by a psychiatrist) according to the Diagnostic and Statistical Manual of Mental Disorders, Fifth Edition (DSM-5).

The schizophrenia inpatient database identified 308 in-patients eligible for participation in the study. Included patients were those who (1) were diagnosed with schizophrenia by a psychiatrist according to the DSM-5 criteria, (2) hospitalized for > 1 year, and (3) clinically stable without a change in the dose of the medications for the last 3 months before the beginning of the study. Five relapse factors, graded as yes/no, were used as a representation for instability criteria in the clinical stability assessment: (1) Occurrence of psychiatric hospitalization [23, 24], (2) Exacerbation or emergence of acute manifestations of the disease [23, 25] (3) Change in medication or a significant increase in the doses [23, 26] (4) Significant suicidal ideation and/or suicide attempt [23, 27] (5) Worsening of the primary mental disorder due to psychoactive substance use/abuse [23, 28]. Clinical stability was defined as the negative answer to the five instability criteria. Patient was considered unstable in case of a yes answer to any of those five criteria [29]. The same selection was followed in a previous study [30].

### Procedures and measures

Patients were assessed before quarantine by a clinical psychologist independent of the study as part of other studies carried out at the hospital. They were then evaluated again by the same psychologist during the quarantine phase. Patients had the right to opt out of the study with such decisions not affecting the treatment offered to them whatsoever.

The questionnaire used during the interviews was in Arabic, the native language of Lebanon. One clinical psychologist was responsible for the data collection via an individual interview with each patient. The first part assessed the sociodemographic and clinical characteristics of the participants (age, sex, education level, marital status, smoking, duration of hospitalizations, knowledge about COVID-19, etc.). The religiosity was assessed by a single question asking about the religious practice (Never/A little bit/Sometimes/Most of the time/All the time). The other parts included the different scales used in this study:

#### Positive and negative syndrome scale

The positive and negative syndrome Scale (PANSS), validated in Arabic language [31], assesses the severity of psychopathology in adult patients with schizophrenia and other psychotic disorders. It’s a 30-item questionnaire, originally organized into separate three subscales: positive symptoms (7 items), negative symptoms (7 items), and general psychopathology (16 items) [32]. All individual items are scored on a scale from 1 to 7 (1 = absence of symptoms and 7 = extremely severe symptoms). Higher scores indicating higher psychotic symptoms. In this study, the Cronbach’s alpha for the positive PANSS was 0.869, for the negative PANSS 0.733, for the general psychopathology 0.754, and for the total PANSS 0.873.

#### Yale-Brown obsessive–compulsive scale

The Yale-Brown obsessive–compulsive scale (Y-BOCS) is a 10-item severity scale that evaluates the time, interference, distress, resistance, and control related to the symptoms. Higher scores indicating more severe obsessive compulsive symptoms [33]. In this study, the Cronbach’s alpha was 0.919. This scale has been previously used in Arabic [34].

#### Calgary depression scale for schizophrenia (CDSS)

CDSS is a nine item structured interview scale that was designed in 1990 specifically to assess depression independently of symptoms of psychosis in schizophrenia [35]. Items are graded on a 4-point Likert type scale (0, absent; 1, mild; 2, moderate; 3, severe). Higher scores indicating the presence of a major depressive episode (Cronbach’s alpha = 0.835). This scale has been previously used in Arabic [36].

#### The World Health Organization quality-of-life (WHOQOL)-BREF

Validated in Arabic [37], this scale evaluates a person’s perception of his QOL and consists of 26 items scored on a five-point Likert scale ranging from 1 (very dissatisfied/very poor) to 5 (very satisfied/very good). Four domains are addressed: physical health (7 items), psychological health (6 items), social relations (3 items), and environment (8 items). Higher scores indicating a higher QOL [38]. The Cronbach’s alpha values were noted as follows: domain 1 (physical health 0.648), domain 2 (psychological health 0.689), domain 3 (social relations 0.589) and domain 4 (environment 0.690) respectively.

### Statistical analysis

Data analysis was analyzed on SPSS software, version 23 [39]. Descriptive statistics included frequencies (percentages) and means (standard deviation) for categorical and continuous variables, respectively. Since the scores did not follow a normal distribution, non-parametric tests were used; the Wilcoxon test evaluated the change in scores between baseline and after 5 months of quarantine. Additionally, a repeated measures ANOVA was performed, aiming to compare symptoms change between baseline and after 5 months of quarantine. A post-hoc analysis using the Bonferroni correction was used to reveal the significant effect of the different symptoms scales used over time; this analysis was adjusted for gender, knowledge about the coronavirus, getting updates about the coronavirus, religion and age. After that, the parameter estimates analysis of the repeated measures ANOVA was used to detect variables related to the dependent variables. Statistical significance was set at *p* < 0.05.

## Results

### Comparison between included and excluded patients

**Fig. 1:**
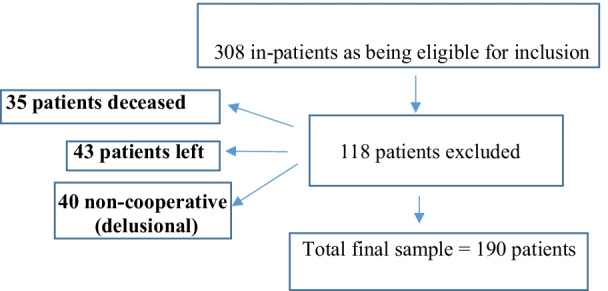
Flow chart of the participants included in the study

A total of 308 patients was screened, of whom 190 patients were included (74.7% men and 25.3% women); 118 patients were excluded for the following reasons: 35 patients died, 43 left, 40 patients’ non cooperative (delusional; unable to answer the questions and to give an informed consent) (See Flow Chart, Fig. 1).

A significantly higher proportion of included patients were males and had a complementary level of education compared to excluded patients. No significant association between the two groups have been found for the other variables (Table 1).

**Table 1:** Comparison between included and excluded participants

	Included	Excluded	
	Frequency (%)	Frequency (%)	
Gender			
Male	142 (74.7%)	58 (49.2%)	< 0.001
Female	48 (25.3%)	60 (50.8%)	
Education level			
Illiterate	12 (6.3%)	8 (6.8%)	0.011
Primary	34 (17.9%)	40 (33.9%)	
Complementary	80 (42.1%)	35 (29.7%)	
Secondary	45 (23.7%)	20 (16.9%)	
University	19 (10%)	15 (12.7%)	
Marital status			
Single	162 (85.3%)	92 (78.0%)	0.253
Married	11 (5.8%)	13 (11%)	
Widowed	1 (0.5%)	2 (1.7%)	
Divorced	16 (8.4%)	11 (9.3%)	
Corona knowledge			
Yes	160 (84.2%)	13 (76.5%)	0.491
No	30 (15.8%)	4 (23.5%)	
Corona updates			
Never	48 (25.4%)	5 (29.4%)	0.553
A little bit	41 (21.7%)	4 (23.5%)	
Sometimes	33 (17.5%)	5 (29.4%)	
Most of the time	60 (31.7%)	3 (17.6%)	
All the time	7 (3.7%)	0 (0%)	
Religion			
Never	23 (12.2%)	1 (5.9%)	0.347
A little bit	35 (18.6%)	2 (11.8%)	
Sometimes	46 (24.5%)	2 (11.8%)	
Most of the time	73 (38.8%)	10 (58.8%)	
All the time	11 (5.9%)	2 (11.8%)	
Family history of psychiatric patients			
Yes	68 (35.8%)	52 (44.1%)	0.152
No	122 (64.2%)	66 (55.9%)	
Age	53.91 ± 10.51	55.26 ± 12.70	0.312
Number of kids	0.33 ± 1.15	0.41 ± 0.93	0.781
Duration of illness in years	26.12 ± 12.59	26.88 ± 12.83	0.608
Duration of hospitalization in years	14.67 ± 10.33	15.94 ± 9.80	0.286

Numbers in bold indicate significant *p* values

The sociodemographic characteristics of the participants are summarized in Table 2. The results showed that the mean age was 54.42 ± 11.40 years with 64.9% males. The majority (82.5%) were single, 21.1% have a secondary level of education, 39.0% have a family history of psychiatric illness and 90.3% were receiving visits from their families before quarantine. Knowledge about the coronavirus has been found in 83.6%. However, following up with the coronavirus updates have been found only in 3.4%. The mean duration of illness and hospitalization were 26.41 ± 12.66 and 15.16 ± 10.13 years respectively.

**Table 2:** Sociodemographic and other characteristics of the patients (N = 190)

	Frequency (%)
Gender	
Male	142 (74.7%)
Female	48 (25.3%)
Education level	
Illiterate	12 (6.3%)
Primary	34 (17.9%)
Complementary	80 (42.1%)
Secondary	45 (23.7%)
University	19 (10.0%)
Marital status	
Single	162 (85.3%)
Married	11 (5.8%)
Widowed	1 (0.5%)
Divorced	16 (8.4%)
Family history of psychiatric patients	
Yes	68 (35.8%)
No	122 (64.2%)
Corona knowledge	
Yes	30 (15.8%)
No	160 (84.2%)
Visits to the hospital	
Yes	171 (90.0%)
No	19 (10.0%)
Corona updates	
Never	48 (25.4%)
A little bit	41 (21.7%)
Sometimes	33 (17.5%)
Most of the time	60 (31.7%)
All the time	7 (3.7%)
Religion	
Never	23 (12.2%)
A little bit	35 (18.6%)
Sometimes	46 (24.5%)
Most of the time	73 (38.8%)
All the time	11 (5.9%)

	Mean ± SD
Age (in years)	53.91 ± 10.51
Length of stay at the hospital in years	15.77 ± 12.04
Duration of illness in years	26.12 ± 12.59
Duration of hospitalization in years	14.68 ± 10.33

Table 3 displays the variation of the measurements of the different scales before and after 5 months of quarantine without adjustment over other covariates. A significant reduction after 5 months of quarantine was reported in the total PANSS scale, the three PANSS subscales (positive, negative and general psychopathology), depression scale and the psychological health quality of life compared to before it (WHO domain—2). However, a significant increase was found in physical health (WHO domain—1) and social relations (WHO domain—3) after 5 months compared to before it. No significant variation was found for the obsession scale and the environment quality of life scale (WHO domain—4).

**Table 3:** Variation of the scales used before and after 5 months of quarantine

	T0	T1	*p* value
	Mean ± SD	Mean ± SD	
Total PANSS	87.41 ± 25.84	66.61 ± 18.55	< 0.001
Positive—PANSS	20.75 ± 10.49	13.73 ± 5.75	< 0.001
Negative—PANSS	21.31 ± 8.48	17.71 ± 5.86	< 0.001
General Psychopathology—PANSS	45.34 ± 12.98	35.16 ± 9.24	< 0.001
YBOCS	0.82 ± 2.22	1.52 ± 5.26	0.263
Calgary scale	5.25 ± 5.07	3.01 ± 3.03	< 0.001
WHO—Domain 1	14.18 ± 2.07	15.10 ± 2.14	< 0.001
WHO—Domain 2	15.02 ± 2.48	13.37 ± 2.32	< 0.001
WHO—Domain 3	9.37 ± 2.68	11.83 ± 3.28	< 0.001
WHO—Domain 4	12.72 ± 1.94	12.65 ± 1.95	0.709

A repeated measure ANOVA was performed taking the scales before and after 5 months of quarantine as dependent variables. The post-hoc tests using the Bonferroni correction revealed that depression and the WHO Domain 4 (environment quality of life scale) remained significant over time after adjustment for covariates (gender, knowledge about the coronavirus, getting updates about the coronavirus, religion and age) (Table 4).

**Table 4:** Effect of time over the measures used adjusted for covariates

Effect	Measure	*df*	Mean square	F	*p* value
*Test of within subject effect*					
Time	Total PANSS	1	476.670	1.761	0.189
	Positive—PANSS	1	6.182	0.130	0.720
	Negative—PANSS	1	51.084	1.278	0.262
	General psychopathology—PANSS	1	148.820	2.325	0.132
	YBOCS	1	37.377	1.415	0.238
	Calgary scale	1	47.380	4.320	0.041
	WHO—Domain 1	1	2.973	1.295	0.259
	WHO—Domain 2	1	0.156	0.054	0.817
	WHO—Domain 3	1	1.502	0.254	0.616
	WHO—Domain 4	1	11.252	4.493	0.038

Adjusted variables: gender, corona knowledge, visits to the hospital, corona updates, religion and age

The multivariable analysis taking the positive PANSS score as the dependent variable, showed that getting updates about the coronavirus most of the times (B = − 4.32, *p* = 0.009) were significantly associated with a decrease in positive symptoms after 5 months of quarantine compared to before it (Table 5, Model 1).

**Table 5:** Multivariable analysis

	Beta	*p* value	95% confidence interval		Partial eta squared
			Lower bound	Upper bound	
*Model 1: Positive PANSS score as the dependent variable*					
Getting updates about coronavirus most of the times versus no*	-4.32	0.009	− 7.52	− 1.13	0.09
*Model 2: Negative PANSS score as the dependent variable*					
Getting updates about coronavirus most of the times versus no*	− 3.64	0.059	− 7.43	0.15	0.05
Getting updates about coronavirus little of the times versus no*	− 3.63	0.071	− 7.59	0.32	0.04
Religiosity all the times versus none of the times*	− 8.02	0.031	− 15.29	− 0.75	0.06
*Model 3: General psychopathology PANSS score as the dependent variable*					
Getting updates about coronavirus most of the times versus no*	− 8.77	0.001	− 13.81	− 3.74	0.14
Getting updates about coronavirus some of the times versus no*	− 5.90	0.048	− 11.76	− 0.05	0.05
Getting updates about coronavirus little of the times versus no*	− 5.67	0.035	− 10.92	− 0.42	0.06
Religiosity all the times versus none of the times*	− 10.50	0.033	− 20.16	− 0.84	0.06
Religiosity some of the times versus none of the times*	− 6.98	0.025	− 13.06	− 0.91	0.06
*Model 4: Total PANSS score as the dependent variable*					
Getting updates about coronavirus most of the times versus no*	− 16.74	0.001	− 26.73	− 6.74	0.13
Getting updates about coronavirus some of the times versus no*	− 10.70	0.071	− 22.33	0.93	0.04
Getting updates about coronavirus little of the times versus no*	− 11.34	0.033	− 21.77	− 0.92	0.06
Religiosity all the times versus none of the times*	− 23.00	0.019	− 42.19	− 3.92	0.07
Religiosity some of the times versus none of the times*	− 13.12	0.034	− 25.19	− 1.05	0.06
*Model 5: WHO Domain 3 score as the dependent variable*					
Gender (females vs. males*)	1.77	0.022	0.27	3.28	0.07

*Reference group

None of the variables was significantly associated with the depression score and the WHO Domains 1, 2 and 4 scores

The results of a second model, taking the negative PANSS score as the dependent variable, showed that practicing religiosity all the time versus none (B = − 8.02, *p* = 0.031) was significantly associated with a decrease in negative symptoms after 5 months of quarantine compared to before it (Table 5, Model 2).

The results of a third model, taking the general psychopathology PANSS score as the dependent variable, showed that getting updates about the coronavirus little (B = − 5.67, *p* = 0.035), some (B = − 5.90, *p* = − 5.90) and most (B = − 8.77, *p* = 0.001) of the times versus none, and practicing religiosity all (B = − 10.50, *p* = 0.033) and some of the times versus not (B = − 6.98, *p* = 0.025) were significantly associated with a decrease in psychopathology symptoms after 5 months of quarantine compared to before it (Table 5, Model 3).

The results of a fourth model, taking the total PANSS score as the dependent variable, showed that getting updates about the coronavirus little (B = − 11.34, *p* = 0.033) and most (B = − 16.74, *p* = 0.001) of the times and practicing religiosity some (B = − 13.12, *p* = 0.034) and all the times (B = − 23.00, *p* = 0.019) compared to none were significantly associated with a decrease in the total PANSS score after 5 months of quarantine compared to before it (Table 5, Model 4).

The results of a fifth model, taking the WHO Domain 3 (social relations) as the dependent variable, showed that female gender (B = 1.77, *p* = 0.022) was significantly associated with an increase in the WHO Domain 3 score (better social relations) after 5 months of quarantine compared to before it (Table 5, Model 5).

It is of note that none of the variables was significantly associated with the depression and the WHO Domains 1, 2 and 4 scores.

## Discussion

As psychiatric patients remain a vulnerable population especially during the COVID-19 quarantine with little research having been conducted on patients with schizophrenia, this study assessed the variation of psychotic symptoms—both positive and negative (PANSS), depression, obsession and quality of life amongst patients with schizophrenia before and after 5 months of quarantine.

Table 3 highlighted significant variation in measures of the different scales before and after 5 months of quarantine without adjustment over other covariates showing reduction in psychopathology and an increase in physical health and social relations after 5 months of quarantine compared to before it. However, obsession and quality of life (domains 1, 2 and 4) had no significant changes. As research during COVID-19 is ongoing, the authors speculate quality of life did not improve within this sample due to quarantine isolating individuals and restricting them from making any life related improvements. This in turn impacted obsession symptoms as individuals were in a current state of anxiety as to what would happen next.

The results of the repeated measures ANOVA focusing on positive, negative, general psychopathology and total PANSS scores all showed significant decrease after 5 months of quarantine which is not in line with the rising rates of mental health related disorders across the globe due to the pandemic [40]. The results are possibly due to the overall symptomatic nature of schizophrenia and how symptoms are expressed. That is, one of the most significant triggers of psychotic symptoms associated with both onset and relapses, is anxiety [41]. Since quarantine has caused increased anxiety and stress worldwide [42] and the developments of COVID-19 such as a cure remains unknown, individuals with schizophrenia fare better by keeping up to date by receiving news both little and most of the times pertaining to COVID-19. Taking a holistic approach with the added value of updating patients about the pandemic, the presence of well-trained staff, therapeutic care and protective measures being administered may all have an impact on reducing fear and anxiety in patients with schizophrenia. It might very well be those patients, during these unprecedented times of a pandemic, feel safe that the standard and care of treatment has not dropped, and their safety has not been compromised.

Positive PANSS, General Psychopathology PANSS score and Total PANSS score were significantly reduced in patients who received constant updates pertaining to COVID-19. Once again, we postulate when anxiety levels are reduced in schizophrenia—in this case fear of COVID-19, positive symptoms also tend to reduce [43]. However, negative PANSS was not affected by receiving updates pertaining to COVID-19 which could be due to the nature of symptomatology of negative symptoms which, on average, requires longer time on treatment before results show a reduction in symptoms [44]. Additionally, while all patients collectively fared better, individuals with higher levels of religiosity and religious practice (i.e., prayer) had the highest significant decrease in general psychopathology, negative PANSS and total PANSS score and we postulate this could be due to the decreased nature of anxiety usually found in religious individuals [45].

### Implications for psychiatric nursing practice

As the COVID-19 crisis is still ongoing and psychiatric hospitals have taken extensive quarantine measures to protect this vulnerable high-risk group, this study highlights the importance of patients being kept up to date with the latest news pertaining to COVID-19 as well as the importance of religiosity as defensive factors against anxiety. While quarantine is important, healthcare professionals (doctors, nurses, etc.) working in psychiatric hospitals/institutions and looking after vulnerable groups, should take into consideration that informing patients constantly about the virus does not leave them in the dark and has the potential to reduce their levels of psychopathology.

### Limitations

While the sample size is geographically representative to the population of Lebanon, the study suffered from methodological limitations. For one, the inclusive criteria only included chronic patients that were hospitalized for more than a year. Secondly, patients were clinically stable and had no changes to their pharmacological regimen. As this study assessed psychosis, depression, obsession, and quality of life during the COVID-19 quarantine, the degree to which updates, and religiosity affected and alleviated these symptoms are unknown. A residual confounding bias is also possible since not all factors associated with the dependent variables were taken into consideration in this study. A selection bias is also possible since all patients were recruited from one hospital, thus, limiting the generalizability of our results. In addition, no comparison was done between included and excluded patients. Finally, the authors did not assess anxiety which has been on the rise globally ever since the pandemic struck. Regardless, as far as the authors are aware of, this is the first cross sectional design assessing symptoms of psychosis, depression, obsession and quality of life in patients with schizophrenia during the COVID-19 pandemic.

## Conclusion

Our study has suggested that patients with schizophrenia fare better symptomatically after 5 months of quarantine if they receive constant updates about COVID-19 and more so if they tended to practice religiosity. The COVID-19 pandemic is associated with stressful impact in our sample since at the time of this manuscript being written, the pandemic is still ongoing. As psychiatric patients are vulnerable, these findings need to be confirmed in future larger population studies. It is important protective measures are being followed vigorously to promote confidence and reassurance to patients that their safety has not been compromised. Finally, as this study assessed patients with schizophrenia during the height of the COVID-19 pandemic in Lebanon during a 5-month period, future studies should assess the long-term impact (12–24 months) on the same patient group in order to examine if these results are a reflection of a short-term reaction to COVID-19. Additionally, as vaccination is the key to combating COVID-19 [46], future studies should also assess the attitude and willingness of patients with schizophrenia to be vaccinated for COVID-19. A study conducted in China assessed two groups on the willingness to pay for the COVID-19 vaccine: one with anxiety and depression and another without any mental disorders. The results showed that both groups were willing to pay for the COVID-19 vaccination but a significantly higher proportion of those with anxiety and depression were more willing to receive the vaccine compared to healthy controls [47]. As the study examined patients with depression and anxiety, it would be worth noting if similar results would be reached if future studies assessed patients with schizophrenia.

## Data Availability

The authors have the right to share the database following a reasonable request to the corresponding author.
